# What people think about fast food: opinions analysis and LDA modeling on fast food restaurants using unstructured tweets

**DOI:** 10.7717/peerj-cs.1193

**Published:** 2023-01-13

**Authors:** Muhammad Mujahid, Furqan Rustam, Fahad Alasim, MuhammadAbubakar Siddique, Imran Ashraf

**Affiliations:** 1 Department of Computer Science, Khwaja Fareed University of Engineering and Information Technology, Raheem Yar Khan, Pakistan; 2 School of Computer Science, University College Dublin, Dublin, Ireland; 3 Department of Industrial Engineering, College of Engineering, King Saud University, Riyadh, Saudi Arabia; 4 Department of Computer Science and Information Technology, Ghazi University, Dera Ghazi Khan, Punjab, Pakistan; 5 Information and Communication Engineering, Yeungnam University, Gyeongsan si, South Korea

**Keywords:** Sentiment analysis, Ensemble model, Latent Dirichlet Allocation, TextBlob

## Abstract

With the rise of social media platforms, sharing reviews has become a social norm in today’s modern society. People check customer views on social networking sites about different fast food restaurants and food items before visiting the restaurants and ordering food. Restaurants can compete to better the quality of their offered items or services by carefully analyzing the feedback provided by customers. People tend to visit restaurants with a higher number of positive reviews. Accordingly, manually collecting feedback from customers for every product is a labor-intensive process; the same is true for sentiment analysis. To overcome this, we use sentiment analysis, which automatically extracts meaningful information from the data. Existing studies predominantly focus on machine learning models. As a consequence, the performance analysis of deep learning models is neglected primarily and of the deep ensemble models especially. To this end, this study adopts several deep ensemble models including Bi long short-term memory and gated recurrent unit (BiLSTM+GRU), LSTM+GRU, GRU+recurrent neural network (GRU+RNN), and BiLSTM+RNN models using self-collected unstructured tweets. The performance of lexicon-based methods is compared with deep ensemble models for sentiment classification. In addition, the study makes use of Latent Dirichlet Allocation (LDA) modeling for topic analysis. For experiments, the tweets for the top five fast food serving companies are collected which include KFC, Pizza Hut, McDonald’s, Burger King, and Subway. Experimental results reveal that deep ensemble models yield better results than the lexicon-based approach and BiLSTM+GRU obtains the highest accuracy of 95.31% for three class problems. Topic modeling indicates that the highest number of negative sentiments are represented for Subway restaurants with high-intensity negative words. The majority of the people (49%) remain neutral regarding the choice of fast food, 31% seem to like fast food while the rest (20%) dislike fast food.

## Introduction

Different fast-food restaurants are currently gaining a lot of attention. Fast food is produced in vast quantities and sold commercially, with a focus on how quickly it can be served. Food that is bought at a restaurant or grocery store, put in a box, and taken home often has parts that have been frozen, cooked, or reheated before consumption. It is becoming increasingly popular to consume meals away from home, and visits to fast-food outlets are expanding at an even more rapid pace. The term ‘fast food’ refers to food that is purchased in dining establishments that do not offer waiter service and instead rely on self-service or carry-out options (Clauson, 2000; Bleich, Wolfson & Jarlenski, 2017). Since the early 1970s, the average number of times per week that Americans consume meals from fast food restaurants has significantly grown. This is probably because fast food is becoming more popular in the U.S. and other developed countries (French, Harnack & Jeffery, 2000). Most of the people who ate at fast-food restaurants said fast food is quick, the taste of food is delicious, enjoyed restaurants while eating food for entertainment and socializing purposes, and for a healthy diet. People also post views on social media platforms (Rydell et al., 2008).

A large part of the population today makes use of social media to express their feelings, reviews, frame of mind, and opinions. People’s public and private opinions on a broad variety of issues are routinely spoken and spread *via* various social media platforms like Facebook, Twitter, Instagram, etc. In terms of popularity, Twitter is one of the most rapidly expanding social networking sites (Saif, He & Alani, 2012). Twitter allows businesses an easy and fast way to gauge how their customers feel about issues critical to their success. The length of tweets is limited to 140 characters. Many companies share their product details on social media platforms, observing and analyzing the public reactions and replying to them on Twitter. With the availability of large bulk of data on social media streams, analysis of such data holds a large potential for valuable knowledge; however, analyzing the reviews manually is not a feasible option anymore. As a result, machine learning-based sentiment analysis emerged as an attractive solution in the past years. Sentiment analysis has strong relationships with both natural language processing and data mining. Many terms can be used to describe sentiment analysis. Subjectivity analysis is also known as appraisal retrieval or information extraction and has implications for human–computer interaction (Pang, Lee et al., 2008).

Natural language processing (NLP) is a field of artificial intelligence that enables machines to perform human-related tasks (Meera & Geerthik, 2022). It is hard for a person to make sense of a large amount of text or numbers by pulling out useful information and knowledge in a short amount of time; so automated processes are needed. In NLP, the main task is to train the machine in such a way that it automatically extracts meanings from the text when a single document contains many similar words with different meanings (Chowdhary, 2020). Hasan, Maliha & Arifuzzaman (2019) developed a framework that contained preprocessed data using NLP and retrieved important tweet text from the data with the help of a bag of words(BoW) and term frequency-inverse document frequency (TF-IDF). Alshamsi et al. (2020) used about 14,000 tweets for sentiment analysis. They cleaned the tweets using NLP techniques and categorized the tweets into positive, negative, and neutral sentiments. Fang & Zhan (2015), solve the issue of polarity classification in sentiment analysis at the sentence level and review level. Another paper (Hazarika et al., 2020) used TextBlob as a part of NLP for performing sentiment analysis on tweets and classifying the polarity score.

### Challenges in sentiment analysis

Sentiment analysis is a robust and efficient NLP technique that can analyze sentiments in a variety of data, including the publicly expressed views and comments of people on social networking platforms. Restaurants can substantially benefit from the automatic extraction of sentiments from large corpora of data (such as sentiments regarding fast food companies) in order to better serve the clients and their recommendations about the food. However several challenges require further research efforts. For example, labeling unstructured tweets using lexicon-based approaches still requires research. Although data cleaning helps to obtain better results, however, it also may cause information loss which is not very well investigated. Lexicon based-methods require extensive preprocessing of tweets to produce more accurate results and the limited availability of sentiment vocabulary across several domains makes it challenging. Similarly, sentiment analysis regarding food is not explored very well. Also, existing methods provide lower accuracy. The proposed method deals with low accuracy, appropriate feature extraction, and multiclass classification problem and provides better results. The BiLSTM+ GRU architecture outperforms single model gated recurrent units (GRUs) and makes the model more robust. The proposed method can reduce ‘variance’, and model bias, and so decreases the possibility of overfitting.

### Contributions

This research aims to investigate how people generally feel about various fast food restaurants, their quality of service, foods, etc., so that more informed, quick, and intelligent decisions can be made by other users. Also, the provision or exclusion of specific food items can be made from restaurants in the light of user reviews sentiments. This will help restaurant owners gain the public’s trust in their restaurants as well as for customers to choose the best restaurant for enjoying the meal. Existing studies predominantly focus on the use of machine learning models for sentiment classification. Accordingly, the performance evaluation of deep learning models in general and deep ensemble models, in particular, are under-explored research areas. This study overcomes these limitations and makes the following contributions.

• A large dataset of unstructured tweets is gathered from Twitter using the Tweepy API regarding the top five fast food companies including McDonald’s, KFC burger, Subway, Burger King, and Pizza Hut. Preprocessing is carried out before applying deep ensemble models to the dataset. For this purpose, NLP is used for preprocessing, data cleaning, and labeling.
• Performance of lexicon-based approaches is evaluated including TextBlob and valency-aware dictionary for sentiment reasoning (VADER). This study makes use of deep learning models including recurrent neural network (RNN), long short-term memory (LSTM), Bidirectional LSTM (BiLSTM), and gated recurrent unit (GRU). It also deploys four customized deep ensemble models including BiLSTM+GRU, LSTM+GRU, GRU+RNN, and BiLSTM+RNN.
• Topic modeling is employed to acquire the most frequently discussed topics and words using the Latent Dirichlet Allocation (LDA) model.

The remaining of this article is divided into four parts. The following section presents the related work. The proposed approach is discussed in the ‘Material and Methods’ section which also includes a brief overview of deep ensemble models and the dataset used for experiments. Experimental results are discussed in the ‘Results and Discussions’ section. Finally, the conclusion is presented.

## Related Work

Due to the popularity of fast food, ease of access, and a large variety of food, fast food restaurants have become prevalent in all kinds of societies. Reviews regarding such restaurants have been used for sentiment analysis, and topic modeling using machine learning and deep learning models.

Sentiment analysis is the most important and widely used approach in the current era for the study of reviews, surveys, and tweets from customers. Different researchers from all over the world have been working on sentiment analysis. A set of Twitter-based sentiments (either positive or negative) about food delivery were extracted with a lexicon-based approach and text-mining (Trivedi & Singh, 2021). A big data analysis-based approach is followed in Ahmed et al. (2021) for online food reviews. Apache spark system is used to analyze the dataset for Amzaon fine food reviews using support vector classifier (SVC), logistic regression, and Naive Bayes. Results indicate that SVC performs better than other models. Trivedi & Singh (2021) consider Swiggy, Zomato, and UberEats online delivery apps. For sentiment analysis using lexicon-based word-emotion, and lexicon-based sentiment classification. Various findings are discussed regarding the positive and negative tweets for the selection of online apps. The tweets related to market information were preprocessed using text-mining and text-preprocessing techniques, and the sentiments were assigned with the TextBlob approach to improve the performance of lexicon sentiments in Ao (2018). Kydros, Argyropoulou & Vrana (2021) analyzed the discussion on Twitter during the coronavirus to determine the sentiments of people towards the pandemic. In Indonesia, Prastyo et al. (2020) examined general and economic reviews of coronavirus tweets. The tweets were then cleaned and divided into two datasets. The first dataset contains only positive and negative sentiments, while the second dataset contains positive, neutral, and negative sentiments. Pokharel (2020) used 615 tweets that were collected from Twitter using the Tweepy python library. The authors removed links, stopwords, spaces, tokens, and punctuation from the tweets and labeled the tweets as positive, negative, or neutral with a TextBlob approach.

Topic modeling has brought new revolutions in the field of text mining and sentiment analysis. It is a technique for mining statistical patterns to uncover hidden meaning in text datasets. Many scholars have published articles on topic modeling. Maier et al. (2018) applied Latent Dirichlet Allocation (LDA) topics to text data and tackled the problems of preprocessing the text data, reliability, and tuning parameters for classifiers and analyzed the resulting topics. The authors extract discussed topics from the text with LDA that better clarified the large corpus. In another study, Jelodar et al. (2019) discussed LDA-based approaches, their applications, and challenges to discover the research development. Bindra (2012) used LDA to investigate social media sites and analyzed them based on topic modeling. Naskar et al. (2016) gathered messages from social sites, particularly Twitter, to analyze user sentiments. The sentiments contain emotions and different topics which are distributed using the LDA approach. Kuang et al. (2021) analyzed the education blogging comments by applying the LDA approach to discover important topics related to school blogging.

For sentiment analysis, both machine learning and deep learning models are extensively employed by several authors. For example, Abdalla & Özyurt (2021) employed three deep learning techniques CNN, BiLSTM, and CNN-BiLSTM for sentiment analysis of fast food companies. The authors used three datasets of 50K, 100K, and 200K tweets for experiments. Results suggest that using a larger amount of data would yield better classification accuracy. BiLSTM obtains the highest accuracy of 95.35% when trained using a 200K dataset. Dashtipour et al. (2021) developed two deep learning models CNN and LSTM that automatically extract important features from the Persian movie comments and classify them into positive or negative sentiments. Experiments show that the LSTM model attains the highest results. The authors used customer feedback and social media data for sentiment classification of customer reviews into three classes in Jain, Kumar & Mahanti (2018). For extracting customer sentiments, the authors used CNN and the LSTM model and compared them with traditional machine learning models. They found that the CNN and LSTM models achieved superior accuracy. Rani & Kumar (2019), trained the CNN model on 50% of movie reviews, and the remaining 50% of reviews are used for testing. The authors achieved 95% accuracy with CNN as compared to ML models. Other studies (Sabba et al., 2022; Ali, Abd El Hamid & Youssif, 2019; Rehman et al., 2019; Yenter & Verma, 2017; Gandhi et al., 2021) also developed deep-learning CNN and LSTM models on movie datasets for sentiment classification. LISAC Laboratory (Elfaik & Nfaoui, 2021) used NLP techniques and the BiLSTM model for sentiment extraction. To extract meaningful and high-quality information from the tweets, they used word2vec with word embedding.

Different deep learning models were employed for sentiment analysis on a Twitter dataset. The authors used several combinations of stand-alone models for sentiment analysis, with Word2vec and Glove for feature extraction and lexical semantics modeling, respectively (Goularas & Kamis, 2019). Ramadhani & Goo (2017) used deep neural networks (DNN) and multilayer perception (MLP) for sentiment analysis. The data are gathered from Twitter and preprocessed before training the models. Results using deep learning models are promising. Another study (Singh et al., 2022) performed sentiment analysis using deep learning models. The proposed method is based on LSTM and RNN and employs attention-layer features in conjunction with preprocessing techniques to make the analysis more effective than existing approaches. The study reported an accuracy score of 94%. Hassan & Mahmood (2018) performed sentiment analysis using a fusion of CNN and RNN approaches. The authors employed pre-trained NLP models for a large dataset. They leveraged CNN to extract features and RNN for sentiment classification. The results demonstrate that the proposed approach can greatly improve classification efficiency.

Abdelgwad (2021) used bidirectional encoder representations from transformers (BERT) model for sentiment classification of Arabic text. They used three datasets: Arabic news, hotel reviews, and human-annotated book reviews for the experiments. The BERT model contains 12 hidden layers, 12 attention-layers, an adam optimizer to optimize the model, a 10% dropout for hotel reviews, a 30% dropout for Arabic news and human-annotated book reviews, and 24, 16, and 64 batch sizes for hotel reviews, book reviews, and Arabic news, respectively. The model is fit with 10 epochs for all datasets. The study achieved 89.510% accuracy on the hotel review dataset with fine-tuning parameters and 59% without tuning the parameters. Chiorrini et al. (2021) used two BERT-based models for sentiment analysis and emotion detection from the tweets. The collected datasets for the experiments are very small and imbalanced, so the authors used an under-sampling technique to balance class samples. Karimi, Rossi & Prati (2021) used the BERT adversarial-training (BAT) model for aspect-based sentiment analysis. Similarly, Prottasha et al. (2022) utilized a BERT model combined with transfer learning to analyze sentiments. The proposed model works well with all word-embedding techniques. A comparative review of existing works is presented in Table 1.

**Table 1:** Results and limitations of related work.

**Authors**	**Method**	**Results**	**Study**	**Limitations**
Abdelgwad (2021)	BERT	The study achieved 30% higher accuracy on hotel review dataset with fine-tuning	Sentiment analysis (SA)	Accuracy is very low for sentiment classification and other performance measures like precision, recall, and F1 score are not mentions in the study to check the robustness of the proposed model. The collected datasets were very limited to training the transformer-based BERT model.
Chiorrini et al. (2021)	BERT	The accuracy of sentiment and emotion analysis using Bidirectional Encoder Representations from Transformers (BERT) models was 92% and 90%, respectively.	Sentiment analysis (SA) and Emotion analysis (EA)	Validation loss for the BERT model was very high as compared to training loss. The BERT model performs well on large datasets. The study used separate models with different parameter values for emotion and sentiment analysis and the number of layers in the BERT model was not utilized in the right way.
Goularas & Kamis (2019)	CNN+LSTM	The study compared different deep learning-models and achieved 59% accuracy.	Sentiment analysis (SA)	The proposed approach results are very low as compared to previous studies mentioned in the paper.
Hassan & Mahmood (2018)	CNN+RNN	The study achieved 93% accuracy on the movie review dataset and 89% on the treebank sentiment dataset.	Sentiment analysis (SA)	Topic modeling is not performed. This is about the movie reviews twitter dataset, not for fast food-related tweets.
Dashtipour et al. (2021)	2D-CNN	The study achieved 89% accuracy on hotel-review dataset.	Sentiment analysis (SA)	Accuracy is not satisfactory and topic modeling is not performed. Also, important lexicon based-approaches like TextBlob and VADER are not used.
Jain, Kumar & Mahanti (2018)	LSTM	The LSTM model achieved 43% accuracy on IMDB and 42% on amazon.	Sentiment analysis (SA)	The results are not good and topic modeling is not performed. Also, the study did not use a combination of different models.
Rani & Kumar (2019)	CNN	The CNN model performed well.	Sentiment analysis (SA)	The authors used a stand-alone CNN model for sentiment analysis. The study did not utilize ensemble or topic modeling.
Elfaik & Nfaoui (2021)	BiLSTM	The proposed approach (novel BiLSTM) achieved outclass results.	Sentiment analysis (SA)	No analysis of food-related sentiment or topic modeling was done in this study.

Most of the studies presented in the related works focus on the sentiment analysis of tweets using deep learning models, and some studies extract only sentiments from the tweets. However, topic modeling is not carried out in such studies. Similarly, several studies do not perform preprocessing which results in poor performance from deep learning and machine learning models. Additionally, the performance of many classification models is very poor, takes a long time (with a higher number of parameters), and is inefficient. Furthermore, there is very limited research on sentiment analysis of fast food companies’ reviews.

To overcome these limitations, we use the Twitter API to collect a large number of tweets concerning the top five fast-food companies and apply NLP to clean, label, and preprocess the tweets. This study used four customized deep learning models. Furthermore, we performed topic modeling to capture the most frequently discussed topics and words from the tweets, which helps businesses and customers plan accordingly.

## Material and Methods

**Figure 1:**
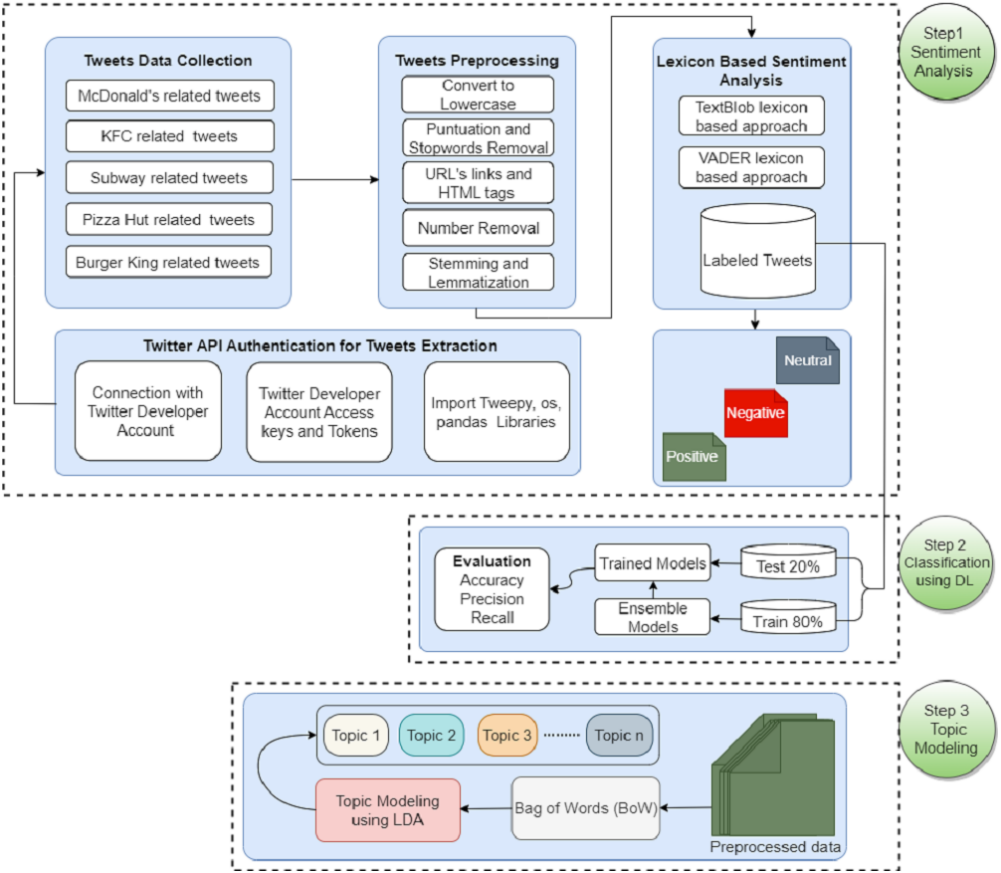
WorkFlow diagram of the adopted methodology for sentiment analysis and topic modeling.

This section discusses the methodology followed for food reviews sentiment analysis for famous fast food companies. Figure 1 shows the flow of the adopted methodology. The data is collected regarding five famous companies including McDonald’s, KFC, Starbucks, Pizza Hut, and Burger King. Tweets are preprocessed to remove unnecessary and redundant information, and lexicon-based sentiment analysis is performed using TextBlob and VADER. Later different deep learning ensemble models are applied for sentiment classification. In the end, LDA is applied for topic modeling.

### Dataset description and preprocessing

We used different hashtags for data collection like #McDonald’s, #KingBurger, #PizzaHut, #Subway, #Subway-sandwich, #KFCburger, etc. to collect a large number of tweets from Twitter using the Tweepy Python library. We collected 14,008 tweets related to McDonald’s, 12,000 related to KFC, 10,861 tweets about Pizza Hut, 14,002 tweets about BurgerKing, and 4,105 tweets about Subway restaurants. The tweets contain ‘user_name’, ‘date’, ‘user_location’, ‘friends’, and ‘text’. The text comprises the tweet and a clickable link to the relevant tweet on Twitter. Our study focused solely on the text variable, with no consideration given to the user or geographic location. The collected raw tweets contain some unnecessary and redundant information and are unlabeled. We used preprocessing steps to filter out irrelevant and redundant data from the tweets (Alzahrani et al., 2022; García, Luengo & Herrera, 2015). Preprocessing is important for efficient training of the models and precise results. First of all, all the tweets are converted into lowercase because the machine takes ‘food’ and ‘Food’ as separate words. Secondly, stopwords are removed from the tweets, and it is most important to remove useless information for improving the performance of models. Stopwords, for example, ‘is’, ‘of’, ‘this’, ‘to’, ‘that’, ‘be’, etc., provide no information about the topic. Also, punctuation like (,? # & []! *) is removed from tweets. However, this does not affect the performance of models. Following that, uniform resource locator (URL) links like ‘https://www.csthi.com’, duplicate text, null values, and numbers are removed from the tweets. In the stemming process, the words are converted into their base form, such as ‘walk’, ‘walked’, or ‘walking’ is transformed into ‘walk’. Details of tweets collected from Twitter are presented in Table 2.

**Table 2:** Number of tweets after and before preprocessing.

**Company**	**No. of tweets before preprocessing**	**No. of tweets after preprocessing**
KFC related tweets	12,000	11,769
McDonalds related tweets	14,008	9,420
Burger King related tweets	14,002	13,732
Pizza Hut related tweets	10,861	6,432
Subway related tweets	4,105	3,772
Total tweets	54,976	45,125

### TextBlob and VADER

TextBlob (Chandrasekaran & Hemanth, 2022) and VADER (Endsuy, 2021) are two of the most widely used lexicon-based approaches that include predefined dictionaries or rules. It makes NLP tasks simple for textual data. Polarity and subjectivity are two outputs received from a sentence when given to TextBlob. Polarity gives two sentiments; −1 for negative sentiment and +1 for positive sentiment. Subjectivity refers to subjects or judgments. TextBlob calculates the sentiments using the Algorithm #1:

**Table:** 

**Algorithm #1: textblob algorithm for sentiments.**
**Input:** Preprocessed tweets (KFC, McDonald’s, Burger King, Pizza Hut and Subway related tweets)
**Output:** Labeled tweets (score > 0 (positive), score < 0 (negative), score = 0 (neutral))
**if** Tweet.sentiment[0] < 0:
sentiment.append(‘Negative’)
**elseif** Tweet.sentiment[0] > 0:
sentiment.append(‘Positive’)
**else** :
sentiment.append(‘Neutral’)
**loop end**

VADER is used for both emotions and positive or negative polarities. It is very smart in NPL tasks, such as it does not require any processing like stopwords, stemming, etc. VADER calculates the sentiments using the Algorithm #2:

**Table:** 

**Algorithm #2: VADER algorithm for sentiments.**
**Input:** Preprocessed tweets (KFC, McDonald’s, Burger King, Pizza Hut and Subway related tweets)
**Output:** Labeled tweets (compound score >= 0.05 (positive), compound score <= −0.05 (negative),
compound score = 0 (neutral))
**if** compound score >= 0.05
sentiment.append(‘Positive’)
** elseif** compound score <= −0.05:
sentiment.append(‘Negative’)
**else:**
sentiment.append(‘Neutral’)
**loop end**

### Ensemble of deep learning-based models

Deep learning is capable of automatically selecting the best features for a variety of tasks. Deep learning is more scalable than machine learning and requires less human intervention. Deep learning models solve classification problems from beginning to end, whereas machine learning needs to split the problems and solve them separately. Deep learning performs better when the dataset size is large. For the current study, the dataset is large and we expect better results using deep learning models, especially deep ensemble learning. In deep learning, overfitting issues can be solved by utilizing dropout layers, batch normalization, regularizers, and removing layers from the model to reduce its complexity. Several studies (Dhola & Saradva, 2021; Rupapara et al., 2021; Onan, 2021) used deep learning for sentiment analysis and showed better accuracy than machine learning models.

Ensemble learning is a useful method for combining the information from more than one conceding model to make the final prediction. It has been argued that ensemble learning would be an effective solution to the problem of overwhelming variance in single prediction models and might help decrease the generalization error (Rokach, 2009). The process of ensemble learning involves the combination of several categorization models rather than the use of a single model to solve a problem. By integrating the layers of several models, the overall performance of these models can become more effective. The four ensemble models are briefly discussed in the subsections below.

### BiDirectional Long Short-Term Memory + Gated Recurrent Unit (BILSTM+GRU)

**Figure 2:**
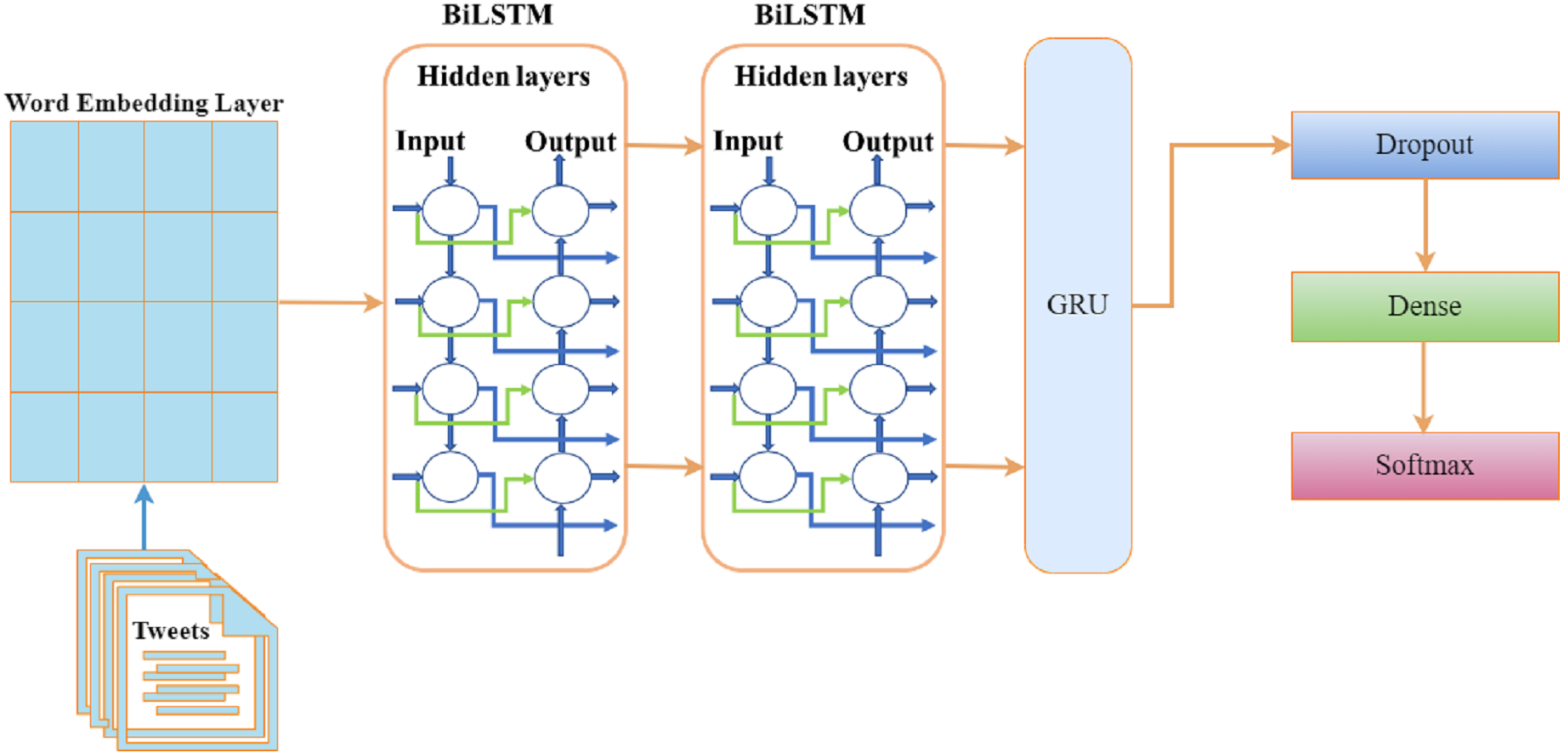
Architecture of the BILSTM+GRU model.

The BiLSTM process sequentially creates neural network information in two directions: past-to-future and future-to-past (Xu et al., 2019). Because of two input directions, a BiLSTM model is different from an LSTM model. We used 8 layers in the ensemble of BiLSTM with GRU. The embedding layer embeds the 6500 ×120 units in the first layer. One dropout of 0.5 is added after the GRU layer to prevent it from over-fitting initially. A GRU model layer with 128 units has been added as the 4th layer. The first dense layer is 64 units, and the second is three units with a softmax activation layer. Our dataset contains three classes, and we used categorical cross entropy as a loss function with the ‘Adam’ optimizer. The architecture of the proposed BILSTM+RU model is depicted in Fig. 2.

### Long Short-Term Memory + Gated Recurrent Unit (LSTM+GRU)

The GRU neural network (Raza, Hussain & Merigó, 2021) simulates the operation of the LSTM. LSTM contains three gates: input, output, and forget, whereas GRU has only the update and reset gates. GRU uses less power than LSTM while having a smaller number of gates. We employed a 7-layer model for the LSTM + GRU ensemble. The 6500 ×120 units in the first layer are embedded in the embedding layer. In this case, 100 and 64 units are used to activate the LSTM layer. There is now a 128-unit GRU model layer available. 32 units in the first dense layer are activated using the l2 regularization kernel known as ‘ReLU’ and three units in the second dense layer are activated using a softmax activation. We utilized the ’Adam’ optimizer and the categorical cross-entropy loss function to evaluate the constructed model on three classes. The architecture of the ensemble model (LSTM+RU) is depicted in Table 3.

**Table 3:** Architectures of Ensemble Deep learning model.

**BiLSTM+GRU**			**LSTM+GRU**		
Layers	Output shape	Param#	Layers	Output shape	Param#
Embedding layer	18,120	780000	Embedding layer	18,120	780000
BiLSTM layer	18,200	176800	LSTM layer	18,100	88400
BiLSTM layer	18,200	176800	LSTM layer	18,64	42240
GRU layer	128	126720	GRU layer	128	74496
Dropout layer	120	0	Dense layer	32	4128
Dense layer	64	8256	SoftMax	3	195
SoftMax	3	99			
**BiLSTM+RNN**			**GRU+RNN**		
Embedding layer	18,120	780000	Embedding layer	18,120	780000
BiLSTM layer	18,200	176800	GRU layer	18,256	290304
BiLSTM layer	18,128	135680	RNN layer	128	49280
Dropout layer	18,128	0	Dropout layer	128	0
RNN layer	128	32896	Dense	64	8256
Dense layer	32	4128	SoftMax	3	195
SoftMax	3	99			

### Gated Recurrent Unit + Recurrent Neural Networks (GRU+RNN)

The RNN model is specially designed for processing sequential information (text, audio, video, etc.). The output is generated to follow the previous samples in the RNN model. It works similarly to that of the GRU, but the operations and gates are different. To solve this, we combined GRU with CNN to enhance the model results. A 7-layer model for the GRU+RNN ensemble with 6500 ×120 units in the first layer is embedded in the embedding layer. In this case, 256 units are used to activate the GRU layer and 128 for simple RNN layers. In the first dense layer, 32 units are activated by the ReLU l2 regularization kernel, and 3 units in the second dense layer are activated by a softmax activation. The architecture of BILSTM+RU is depicted in Table 3.

### BiDirectional Long Short-Term Memory + Recurrent Neural Network (BILSTM + RNN)

We used an 8-layer model for the BiLSTM + RNN ensemble. The 6500 ×4120 units in the first layer are embedded in the embedding layer. 100 units are used to activate the first LSTM layer and 64 for the second. A 0.5 unit dropout is added in the 4th layer. 32 units in the first dense layer are activated using the l2 regularization kernel and 3 units in the second dense layer are activated using a softmax activation. We utilized the Adam optimizer and the categorical cross-entropy loss function to evaluate the constructed model. The architecture of the BILSTM+RU model is depicted in Table 3.

### Transformer based models

Recently, NLP witnessed rapid growth led by pre-trained supervised models. Such models contain hundreds of millions of parameters and show very good performs for NLP tasks. Particularly, the BERT model has achieved an iconic position recently. These models are significant advancements over their previous models (Devlin et al., 2018). In 2018, Google’s AI team developed a model for sentiment analysis (SA), text prediction (TP), and question-and-answer tasks called BERT. The BERT attention mechanism is trained to recognize associations between words in a phrase based on their context. The encoder receives input text and the decoder outputs it, but the encoder mechanism is extremely crucial.

BERT is one of the most comprehensive models for NLP and several variants have been proposed. It has a large size and a high number of parameters which makes it effective. However, it requires large computation time which inhibits the model’s ability to operate quickly and efficiently on low-power devices. Similarly, it has reliability problems on low-powered devices. In some real-time scenarios, any accuracy gains from an improvement may be overturned if the system’s response time is too slow. To reduce the time required to execute the model’s computations, several modifications have been introduced.

The BERT model is complex and hard to train model and may take days for training depending on the size and nature of the data. On the other hand, XLNet transformer-based auto-regressive, as well as the auto-encoding model achieved state-of-the-art performance in many NLP applications. Similar to BERT, XLNet uses bidirectional text and avoids the limitations posed by masking in the BERT model. The XLNet manages to capture a greater number of dependency pairs than BERT. Both models are used in this study for experiments with the hyperparameters given in Table 4.

**Table 4:** Trainable parameters for BERT and XLNet models.

**Model**	**Trainable parameters**	**Hyper-parameters values**
BERT	109,495,187	Learning rate = 0.001, Optimizer = Adam Batch Size = 16
XLNet	116,722,181	Learning rate = 5e−05, epsilon = 1e−08, Optimizer = Adam Batch Size = 16

In this study, sentiment classification was also performed using the BERT model. We used 400 words from each text as input layers, a Keras layer with 109,482,241 parameters, two dense layers with units of 16 and 32, one for multiclass classification, and two dropout layers with units of 0.5 and 0.2.

### LDA based topic modeling

Topic modeling aims at finding a group of similar words or topics which are frequently discussed in tweets. Contrary to the classification of sentiments from machine learning models which show only the type of sentiments, topic modeling provides frequent words used in the text. In the context of food-related tweets, it provides the positive and negative aspects of food companies’ menus and services which can be used by the companies to improve the quality of services.

**Figure 3:**
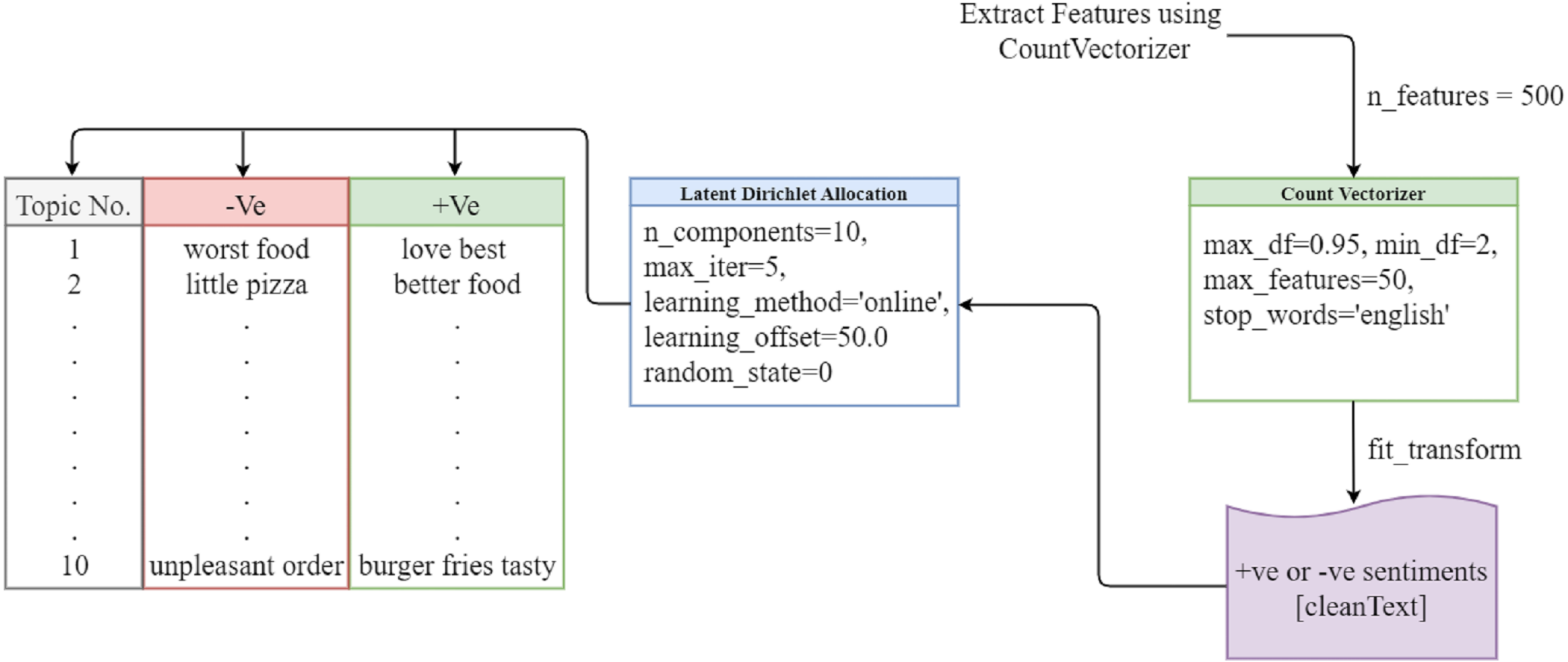
LDA topic modeling.

The LDA model has become the most researched and extensively used model in many disciplines since it addresses the shortcomings of other models such as latent semantic indexing (LSI), latent semantic analysis (LSA), and probabilistic latent semantic indexing (PLSI). LDA classifies, interprets, and evaluates a large collection of textual information, exposing hidden topics in the process (Yang & Zhang, 2018). It divides the topic into different topics and divides the large documents into smaller dimensions. When we do this manually, it will take a long time, but the LDA model can extract this in a very short time and play important role in sentiment analysis. The words assigned to the topic and their distribution according to the Dirichlet distribution. The LDA model provides the best results. The LDA model is shown in Fig. 3.

We use a bag of words to extract features from the cleaned tweets, setting the number of features to 500, the maximum number of features to 50, and the stop words to English. Then, we fit countVectorizer to the clean text data containing sentiment values and apply the LDA model with 10 components and a maximum of five iterations. The LDA model then extracts relevant topics.

### Evaluation metrics

The performance of the models is evaluated using different evaluation metrics (Hossin & Sulaiman, 2015). The evaluation is performed using the test data to check the performance of models according to their efficiency. Accuracy, precision, and recall parameters are used in this study. Accuracy is determined by dividing true positive (TP) plus true negative (TN) predictions over total predictions. Precision is determined by dividing TP predictions divided by TP plus false positive (FP) predictions. The recall is computed by dividing TP predictions over TP plus false negative (FN) predictions.

## Results and Discussion

In this section, experimental results (sentiments of the public towards fast food restaurants) of the lexicon-based approach, the ensemble of deep learning models, and discussed topics using the LDA approach are presented and discussed.

### Sentiment analysis using lexicon-based approaches

The two most widely used lexicon-based approaches are used for sentiment analysis of all tweets like McDonald’s tweets, KFC tweets, etc. Table 5 represents the sentiment of the public towards fast food-related tweets using two lexicon-based approaches. All tweets contain mostly neutral sentiments. Tweet sentiments related to different fast food restaurants using the TextBlob approach are represented. A total of 3,253 tweets are used for positive sentiments and another 2,453 are negative sentiments about KFC. Public sentiments towards McDonald’s are 4,616 neutral, 2,952 positive, and 1,852 negative as shown in Table 5. There are 3,343 neutral tweets, 2,012 positive tweets, and 1,077 negative tweets about Pizza Hut. Following that, Burger King and Subway restaurant tweets, people have 7,048 and 1,558 neutral sentiments, 3,896 and 1,388 positive sentiments, and 2,788 and 1,026 negative sentiments, respectively. Using the VADER approach, the public shows mostly neutral sentiments about different restaurant foods, as in TextBlob sentiments. People have 2,424 sentiments against the fast food service and quality out of 11,769 towards KFC. Only 3,280 sentiments have positive emotions towards McDonald’s.

**Table 5:** Sentiments extracted from top five fast food restaurants using Lexicon-Based Approaches.

**Datasets**	**TextBlob approach**			**VADER approach**		
	Positive	Negative	Neutral	Positive	Negative	Neutral
KFC tweets	3,254	2,354	6,063	4,047	2,424	5,298
McDonald’s tweets	2,952	1,852	4,616	3,280	2,350	3,790
Pizza Hut tweets	2,012	1,077	3,343	2,452	1,128	2,852
Burger King tweets	3,896	2,788	7,047	4,735	3,100	5,897
Subway tweets	1,388	1,026	1,558	686	2,440	846

Results reveal that using the VADER, the number of positive sentiments is higher as compared to using TextBlob except for Subway food sentiments where the positive sentiments using TextBlob are higher. It can be observed that although the same tweets are used for sentiment analysis using TextBlob and VADER, the sentiments assigned by the two are substantially different.

Table 6 displays the most commonly used words identified in the tweets. Good, love, better, best, gift, start, pizza, hut, kfc, burger, happy, customer, sandwich, order, win, etc are words that are mostly used for positive sentiments, and words like bad, sorry, fuck, dead, shit, food, please, unpleasant are treated as negative sentiments in the tweets.

**Table 6:** Most common words in fast food tweets with their classified sentiments.

**Company name**	**Most common words**	
	**Positive sentiments**	**Negative sentiments**
KFC	good, love, like, better, best, really, new, first, get, kfc	Fries, bad, sorry, popeyes, got, order, shit
Pizza Hut	Gift, love, start, pizza, hut, good, win, card	Pizzahut, unpleasant, sorry, please, share, hut, little, bad
McDonald’s	macdonalds, good, like, better, happy, really, right, meal, kids	shit, got, fucked, fucking, water, food, nuggets
Burger King	burger, king, like, good, food, day, love, got, one, better	behind, wall, mall, found, vintage, Delaware, intact, employee, restaurant
Subway	employee, customer, shooting, order, sandwich	Sandwich, much, mayo, shoot, killed, Atlanta, police, dead

**Table 7:** BERT and XLNet results using TextBlob and VADER approaches.

Model	Accuracy	Class	Precision	Recall	F1 score
	**TextBlob approach**				
BERT	95.41	Positive	95	95	95
		Negative	92	92	92
		Neutral	97	97	97
XLNet	94.12	Positive	94	93	94
		Negative	90	90	90
		Neutral	96	96	96
	**VADER approach**				
BERT	93.45	Positive	95	92	94
		Negative	87	93	91
		Neutral	97	93	95
XLNet	93.54	Positive	95	93	94
		Negative	93	90	91
		Neutral	93	96	95

### Results of transformer-based models

The BERT model was used by many researchers to classify the sentiments. Abdelgwad (2021) used the BERT model for sentiment classification on the hotel-review dataset, fitted the model on 10 epochs, and achieved an 89.50% accuracy by employing 10% dropout layers and 24 batch sizes. Singh, Jakhar & Pandey (2021) used the BERT model for emotion classification on Twitter data and attained 93.80% accuracy. Similarly, Pota et al. (2021) compared the performance of the BERT model with the proposed model which was trained using five epochs with an eight-batch size. Results showed that the performance of BERT is superior. In view of the reported accuracy of the BERT model, this study also employs BERT for experiments. Since training a BERT model requires a lot of processing power, most studies only use 5, 8, or 10 epochs. We trained the BERT model using 10 epochs.

The results of the BERT model using the TextBlob and VADER approaches are given in Table 7. Using TextBlob features and VADER features extracted from fast food tweets, the BERT model achieved 95.41% and 93.45% accuracy scores, respectively. TextBlob features led the BERT model to obtain 97% precision, 95% recall, and 92% F1 score on neutral tweets, 95% on positive tweets, and 92% on negative tweets. Using the VADER features, precision, recall, and F1 scores are low.

The results of the TextBlob and VADER-based XLNet model are also included in Table 7. The XLNet model obtained an accuracy of 94.12% and 93.54%, respectively, when provided with TextBlob and VADER features, respectively. Recall scores of 96% for neutral tweets and 90% for negative tweets are achieved by the XLNet model using TextBlob and VADER, respectively. The XLNet model outperforms the BERT model in terms of accuracy and precision on VADER features.

### Results of ensemble deep learning models

**Figure 4:**
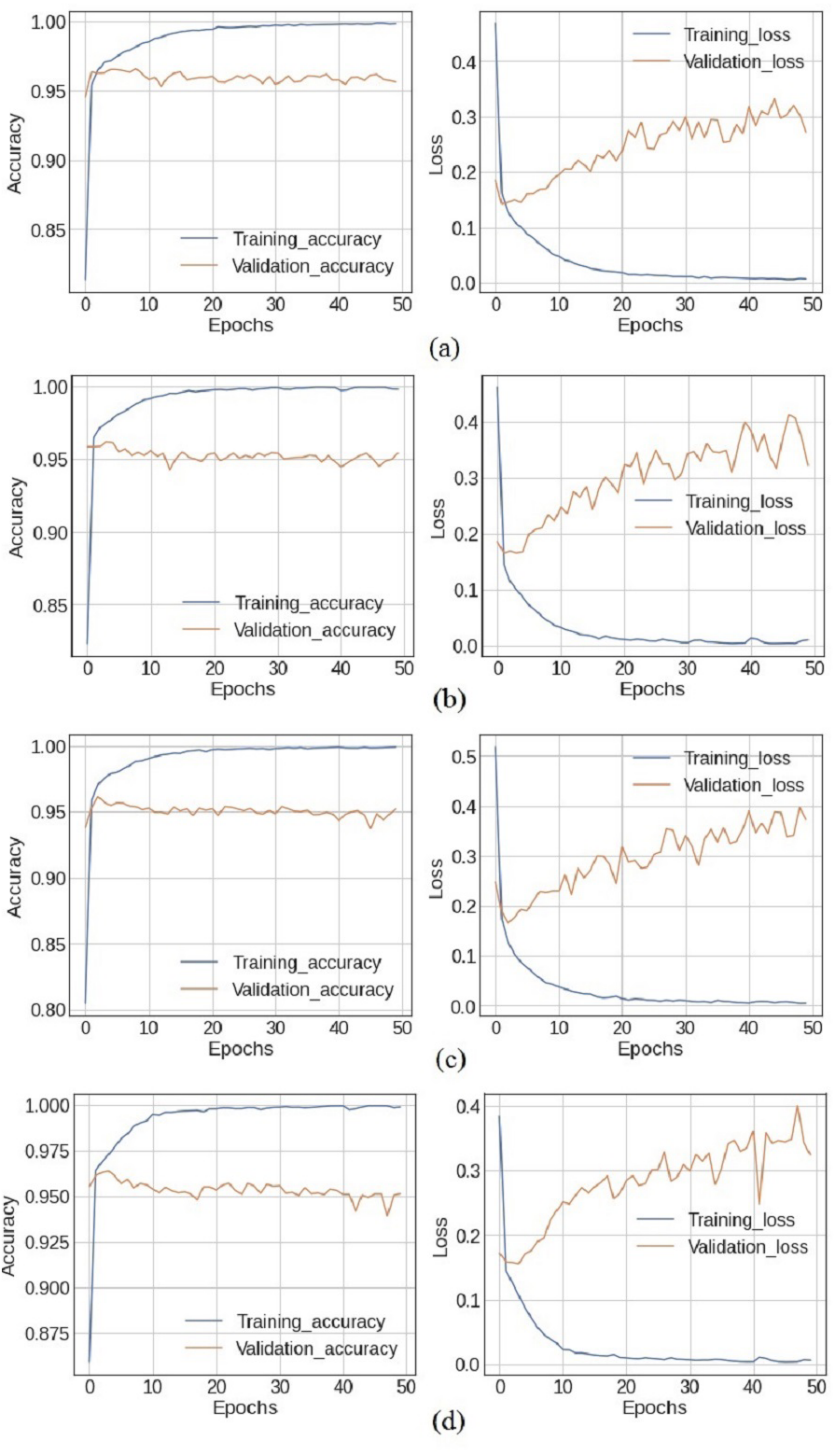
The *X*-axis shows number of epochs and *Y*-axis shows accuracy and loss curves (training_loss,training_accuracy, validation_accuracy, validation_loss) of (A) BiLSTM+GRU model, (B) LSTM+GRU model, (C) BiLSTM+RNN model and (D) GRU+RNN model.

Experiments are performed using both the standalone deep learning models, in addition to the designed ensemble models. Figure 4 shows the training and loss curves of ensemble deep learning models. At epochs 40, 41, and 46, the training accuracy is at its highest with 99.92% accuracy, while the validation accuracy is at 95.62% using the BiLSTM+GRU model. In the LSTM+GRU model, the highest training accuracy of 99.93% is at epochs 45, while the validation accuracy is 95.41%. The training accuracy and loss curves increase after epoch 20 and the highest values are obtained at epoch 41 for all the models.

The ensemble deep learning models, BilSTM+GRU, GRU+RNN, LSRM+GRU, and BiLSTM+RNN perform better as compared to individual models, which are listed in Table 8. The simple RNN model used 816,099 parameters for training the model and obtains a 93.15% accuracy with the TextBlob approach and 89.3% accuracy with the VADER approach. The other three models LSTM, BiLSTM, and GRU are trained with 912,819, 677,027, and 880,227 parameters, and they got 94.67%, 94.63%, and 94.63% accuracy, respectively with TextBlob. The ensemble BiLSTM+GRU achieved 95.62% accuracy which is better as compared to any single model.

**Table 8:** Performance of deep learning standalone and ensemble models.

**Models**	**Parameters**	**Accuracy**	
		**TextBlob**	**VADER**
RNN	816,099	93.15	89.31
LSTM	912,819	94.67	90.05
BiLSTM	677,027	94.63	89.89
GRU	880,227	94.63	90.18
BILSTM+GRU	1,332,771	95.62	90.81
GRU+RNN	1,128,035	94.87	91.37
LSTM+GRU	989,363	95.41	90.49
BILSTM+RNN	1,129,603	95.20	90.59

The precision, recall, and F1 score of different standalone and ensemble models are presented in Table 9 using extracted features from the TextBlob approach. The individual model RNN achieved very low precision, recall, and F1 score on positive tweets, but 89% precision, 86% recall, and 87% F1 score on negative tweets. The GRU achieved a 94% overall score on positive tweets, with 91% precision, an F1 score, and a 92% recall score. Both the ensemble BILSTM+GRU and BILSTM+RNN models achieved 97% precision, recall, and F1 scores which shows the superiority of ensemble models for sentiment classification for food-related tweets.

**Table 9:** Precision, recall, and F1 score of standalone and ensemble models using TextBlob features.

**Model**	**Class**	**Precision**	**Recall**	**F1score**
RNN	Positive	91	91	91
	Negative	89	86	87
	Neutral	96	97	97
LSTM	Positive	94	94	94
	Negative	91	92	92
	Neutral	97	96	97
BiLSTM	Positive	94	94	94
	Negative	92	91	91
	Neutral	96	96	96
GRU	Positive	94	94	94
	Negative	91	92	91
	Neutral	97	96	96
BiLSTM+GRU	Positive	95	95	95
	Negative	93	93	93
	Neutral	97	97	97
GRU+RNN	Positive	95	94	94
	Negative	93	90	91
	Neutral	97	97	97
LSTM+GRU	Positive	94	95	94
	Negative	93	92	93
	Neutral	97	97	96
BiLSTM+RNN	Positive	93	95	94
	Negative	93	92	91
	Neutral	97	97	97

Table 10 presents the precision, recall, and F1 score of standalone and deep ensemble models using the VADER approach. Similar to its performance with the TextBlob, the RNN model also achieved a low score on positive, negative, and neutral tweets using VADER. Individual BiLSTM models achieved an F1 score of 81% on negative tweets, while BiLSTM+RNN and BiLSTM+GRU achieved the highest F1 score of 87%. The GRU+RNN achieved an 89% F1 score and a 93% recall score. Both TextBlob and VADER in Tables 9 and 10 showed that the ensemble models perform better than the standalone models.

**Table 10:** Precision, recall, and F1 score of standalone and ensemble model using VADER features.

**Model**	**Class**	**Precision**	**Recall**	**F1 score**
RNN	Positive	90	89	89
	Negative	83	84	84
	Neutral	92	94	93
LSTM	Positive	91	91	91
	Negative	86	86	86
	Neutral	93	91	92
BiLSTM	Positive	91	90	91
	Negative	87	86	81
	Neutral	91	92	91
GRU	Positive	90	90	90
	Negative	88	88	88
	Neutral	92	93	93
BiLSTM+GRU	Positive	91	92	92
	Negative	88	88	87
	Neutral	92	92	92
GRU+RNN	Positive	92	92	92
	Negative	89	88	89
	Neutral	92	93	93
LSTM+GRU	Positive	90	92	91
	Negative	86	88	87
	Neutral	94	91	92
BiLSTM+RNN	Positive	92	91	91
	Negative	87	87	87
	Neutral	92	92	92

### Topic extraction using LDA topic modeling

Topic modeling (Mujahid et al., 2021) in text-mining is used to understand the large corpus of text and provide useful insights about the topics discussed in the text. For topic modeling, LDA (Farkhod et al., 2021) is used on the preprocessed data and vectors created from the BoW. Then, we extract 10 highly discussed topics from the document that contains the keywords. Topic modeling intends to categorize the text into documents and words. Each topic includes a research-related keyword and a weighting. The positive and negative results of topic modeling based on the LDA approach are presented in Table 11.

**Table 11:** Highly discussed topics using LDA topic modeling on McDonald’s tweets.

**Topic No.**	**Positive keywords**	**Negative keywords**
1	mcdonald love best mcdonalds amp make hot kids eat today	want mcdonald chicken meal fries man fucking day eating order
2	good mcdonald eating time thing better getting today man know	little mcdonald old year girl shot said threw water employee
3	time mcdonald burger free year great getting big mcdonalds make	eat bad mcdonald got food day fuck getting meal want
4	really mcdonald mcdonalds great eating man think right way like	mcdonald people man crazy kids eat mad shit getting got
5	food fast mcdonald need think want sure people burger love	getting order mcdonald amp employee fries water meal like wrong
6	mcdonald fries real drive better food fast today know time	really food nuggets time think fries fucking meal want work
7	mcdonald got thing hot large lmao eat like make eating	mcdonald bad hate know eat really says wrong went want
8	today work mcdonald got happy kids new really meal getting	fries mcdonald eat got kids went year old wrong fucking
9	mcdonald way big better great lol drive sure hot real	chicken mcdonald nuggets eat order meal shit time old want
10	mcdonald right sure make getting happy eating burger kids eat	meal day food mcdonald want sprite got know chicken drive

**Figure 5:**
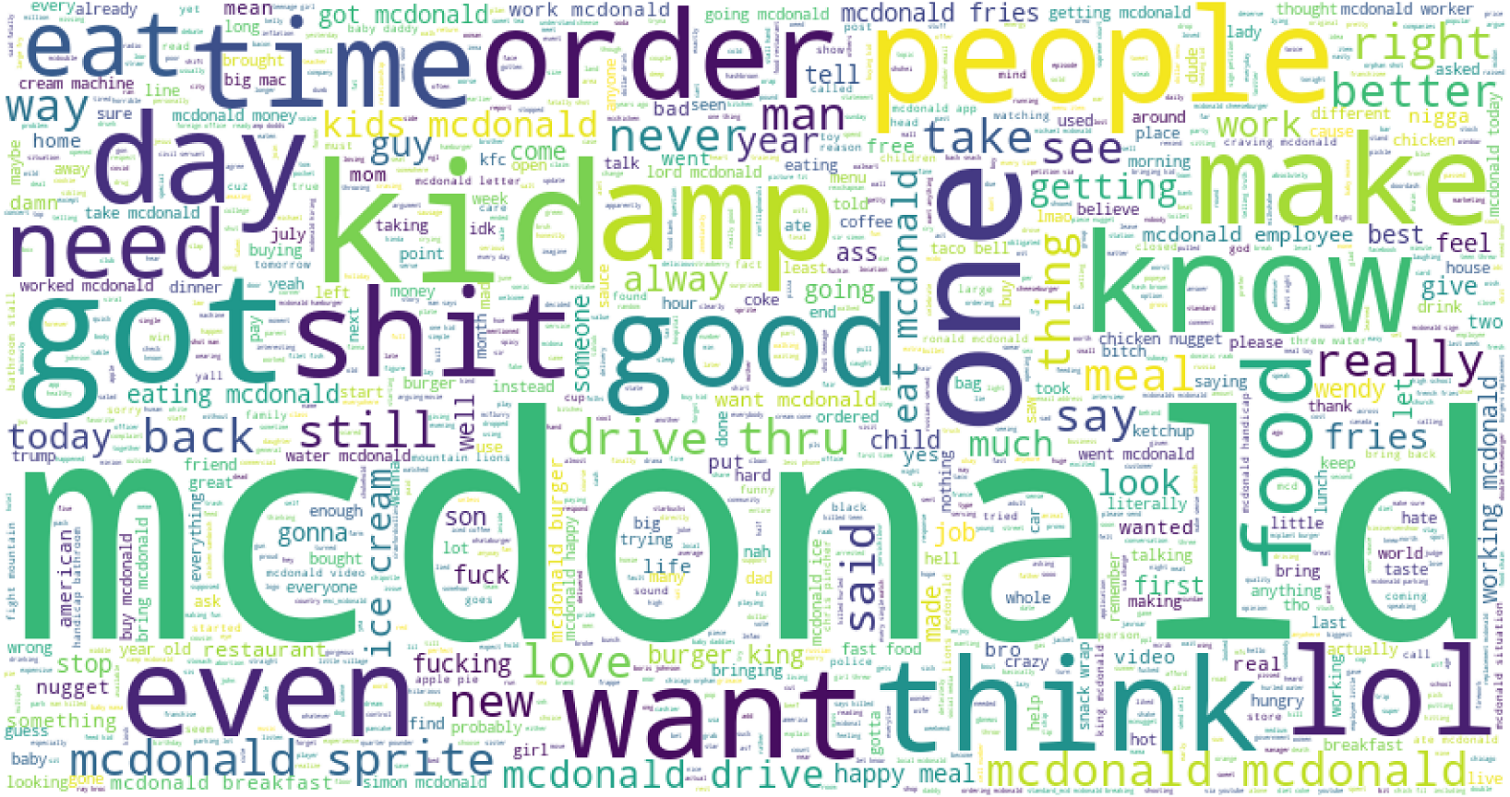
The word cloud for McDonald’s tweets.

Table 11 presents the most frequently discussed topics in McDonald’s tweets. The favorable words show that kids love to eat fast food at McDonald’s. Today, McDonald’s fries really drive better food fast. Kids are very happy to eat burgers and fries. The negative sentiments like kids eat fries at Mcdonald is year old wrong, chicken mcdonald nuggets meal order not arrive in time, a little mcdonald old girl shot because of threw water on employee. These are topics extracted from the McDonald’s tweets. Figure 5 shows the word cloud of the most frequently used words in tweets for McDonald’s. Table 12 presents the most common discussed topics in KFC tweets. The negative sentiments are: really bad fried chicken, sandwiches and fries are fucking India, KFC, hate chicken burger, sorry contact number worst hear, etc.

**Table 12:** Highly discussed topics using LDA topic modeling from KFC-related tweets.

**Topic no.**	**Positive keywords**	**Negative keywords**
1	kfc amp day order going chicken happy time food thing	meal kfc_uki fucking kfc chicken fried really sorry like hear
2	kfc better best really know time hot want bucket chicken	recipe eating hear mean hard bad man contact fuck thin
3	food kfc fast meal jack eating like want try real	eating little kfc meal said sandwich like fucking fries kfc_india
4	kfc got try kfcsa team expressoshow gatsby like best want	eat kfc fries chicken want bad burger fucking think meal
5	kfc_es lmao old sure think time good popeyes amp lol	kfc bad popeyes want chickens everyday said time people day
6	kfc love lol right think happy people really know new	hate kfc really fucking like kfc_india meal sorry day hear
7	kfc new popeyes man better make sure think amp try	like kfc shit hard closed chicken bad time got mean
8	nice lol thing new kfc want going like better got	think kfc sorry man hear worst wrong contact number really
9	kfc good like free great thing sure chicken	wtf kfc_es like man burger hate chicken going
10	kfc real eat live burger people kfc_uki good man	sorry hear kfc contact number said mean know want

**Figure 6:**
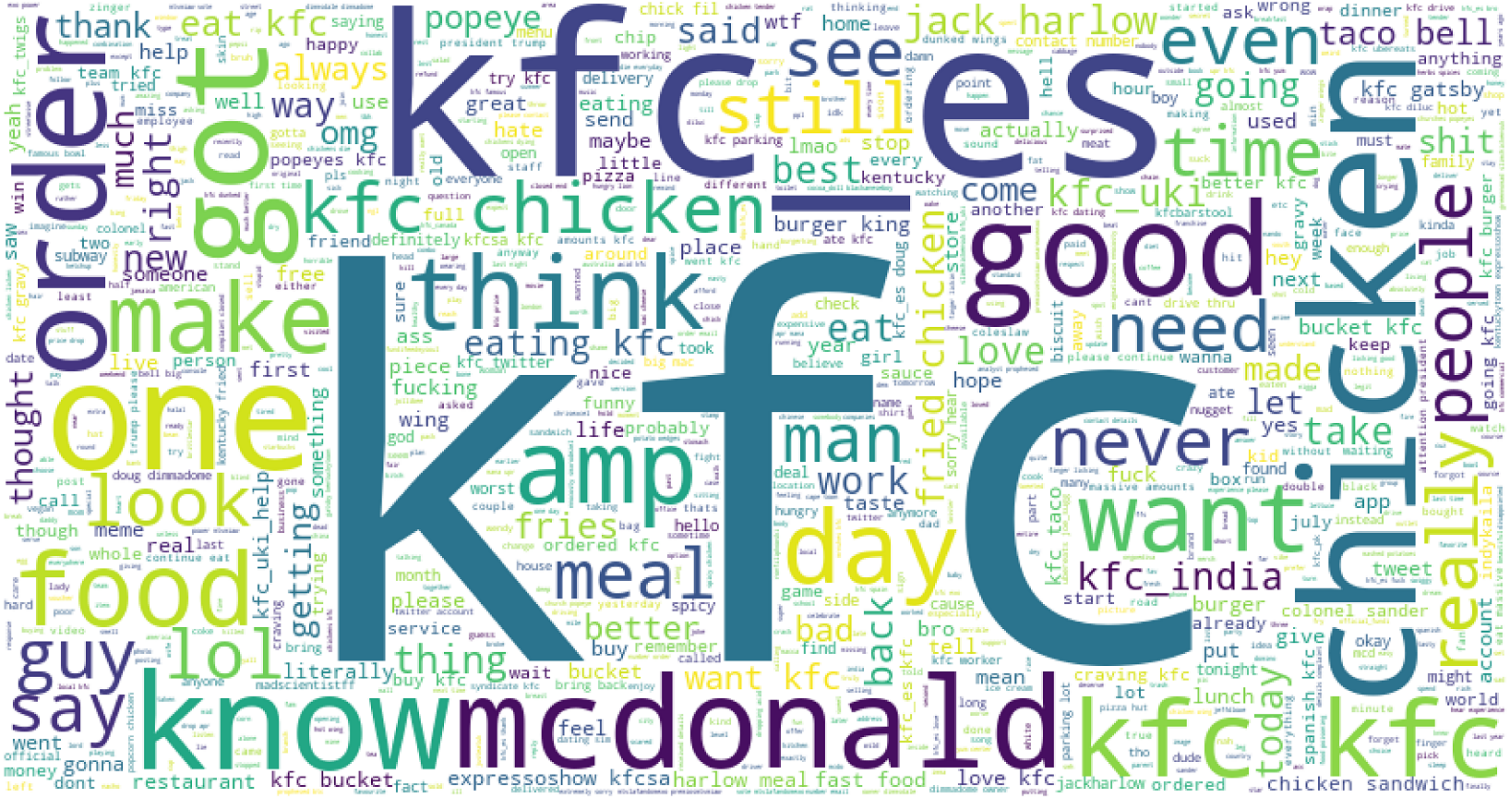
The word cloud for KFC tweets.

Figure 6 shows the word cloud for KFC tweets. LDA extracted the most common words from Burger King-related tweets are presented in Table 13. Burger King-related main positive and negative words are listed here. It shows that tweets use several bad words in negative sentiment-containing tweets like ‘bad amp work’ ‘hate time’, ‘shit bag’, ‘fucking white got black’, etc. It shows that negative words are more frequent than good words.

**Table 13:** Frequently discussed topics using LDA technique from Burger King-related tweets.

**Topic no.**	**Positive keywords**	**Negative keywords**
1	right burger king fries best amp gift fast good job	burger king bad amp work chicken bag day eat fries
2	mall old restaurant viral working new burger king know eat	got went burger king hate time shit bag eat restauran
3	food fast want viral like new mall kevin king burger	way burger king people chicken fries man intact mall shit
4	burger king right free amp eat fast way mcdonald know people	king burger day know great mall really like working white black years going eat hate shit receives working know
5	burger king know way got good really sure amp best	burger king eat whopper impossible know fucking white got black
6	right eat mcdonalds free king kevin burger good work bag	man black burger king like hate bag work white chicken
7	best getting working fries burger king mall man think viral	burger king food working missing years bag like black day
8	good new got video viral work lol donations know great	years burger king employee gift work mediocre viral receives gofundme
9	job viral video king burger gift getting like working lol	burger king chicken fries long white way realjezebelley know wall
10	fast kevin really working way day years love king donations	fucking shit burger king like mcdonald going hate food bag

**Figure 7:**
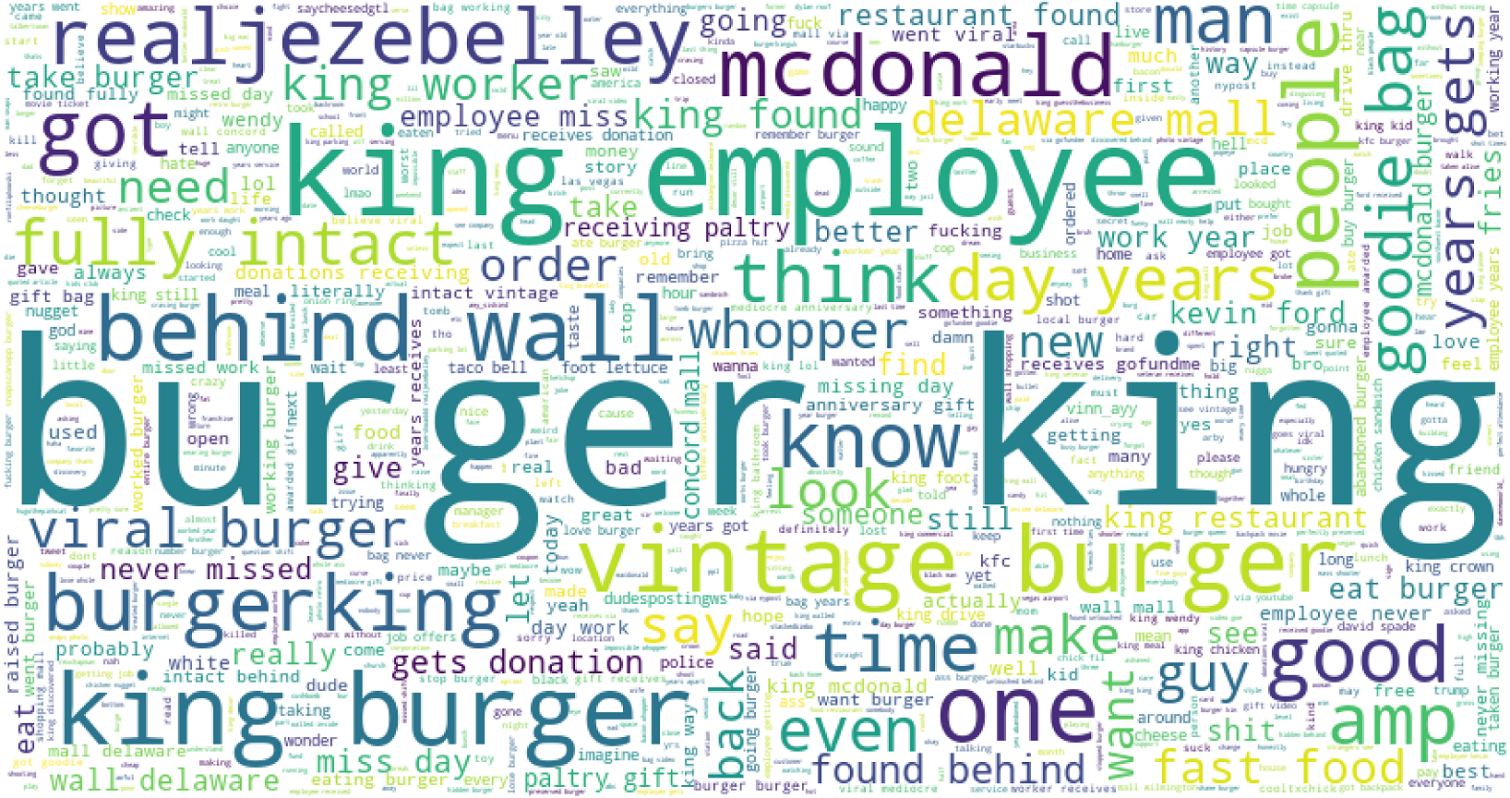
The word cloud for frequently used words for Burger King.

The word cloud for the most frequently used words in Burger King tweets is given in Fig. 7. The word cloud shows that several words are frequently used like ‘king emplouee’, ‘king burger’, ‘viral burger’, ‘behind wall’, etc.

Table 14 shows that Pizza Hut started a short survey to win a gift card for pizza domino’s, and it is better for the company. Pizza Hut had the same disgusting thing about three years ago. Pizza Hut should bring back the Sicilian-style pizza they had back in 1998. I ordered Pizza Hut and Wing Stop to be delivered at the same time. This is going to be so embarrassing. I could crush some Pizza Hut wings; apparently, there’s a Pizza Hut in my city now, so God bless the United States.

**Table 14:** Extracted frequently used words from Pizza Hut-related tweets.

**Topic no.**	**Positive keywords**	**Negative keywords**
1	win card gift hut pizza chance short start survey know	pizza hut like little caesars domino papa dominos shit kfc
2	sure day favorite food like pizza hut got know reall	domino little pizza email caesars fuck share unpleasant number hut
3	pizza good hut dominos pizzas papa got little amp like	pizzahut order worse pizza ordered bad hut restaurant domino time
4	love eat favorite survey gift taco know right used time	really phone hut sorry pizza share email unpleasant experience number
5	start short survey win gift chance card hut pizza school	sorry number unpleasant ordered food experience email used phone little
6	pizza hut better know domino remember way papa think like	really dominos papa used time eat experience order want worst
7	eat pizza hut lol know school amp like food win	pizza hut bad got fuck want worst food kfc know
8	pizza hut like really team better want right amp going	crust ordered pizza amp hut wings worse shit chicken used
9	used pizza food hut papa better survey card start chance	pizza hut eat time wrong cheese fucking used ordered amp
10	old pizza hut years school like got amp used better	ant domino cheese food shit hut night dominos really fucking

**Figure 8:**
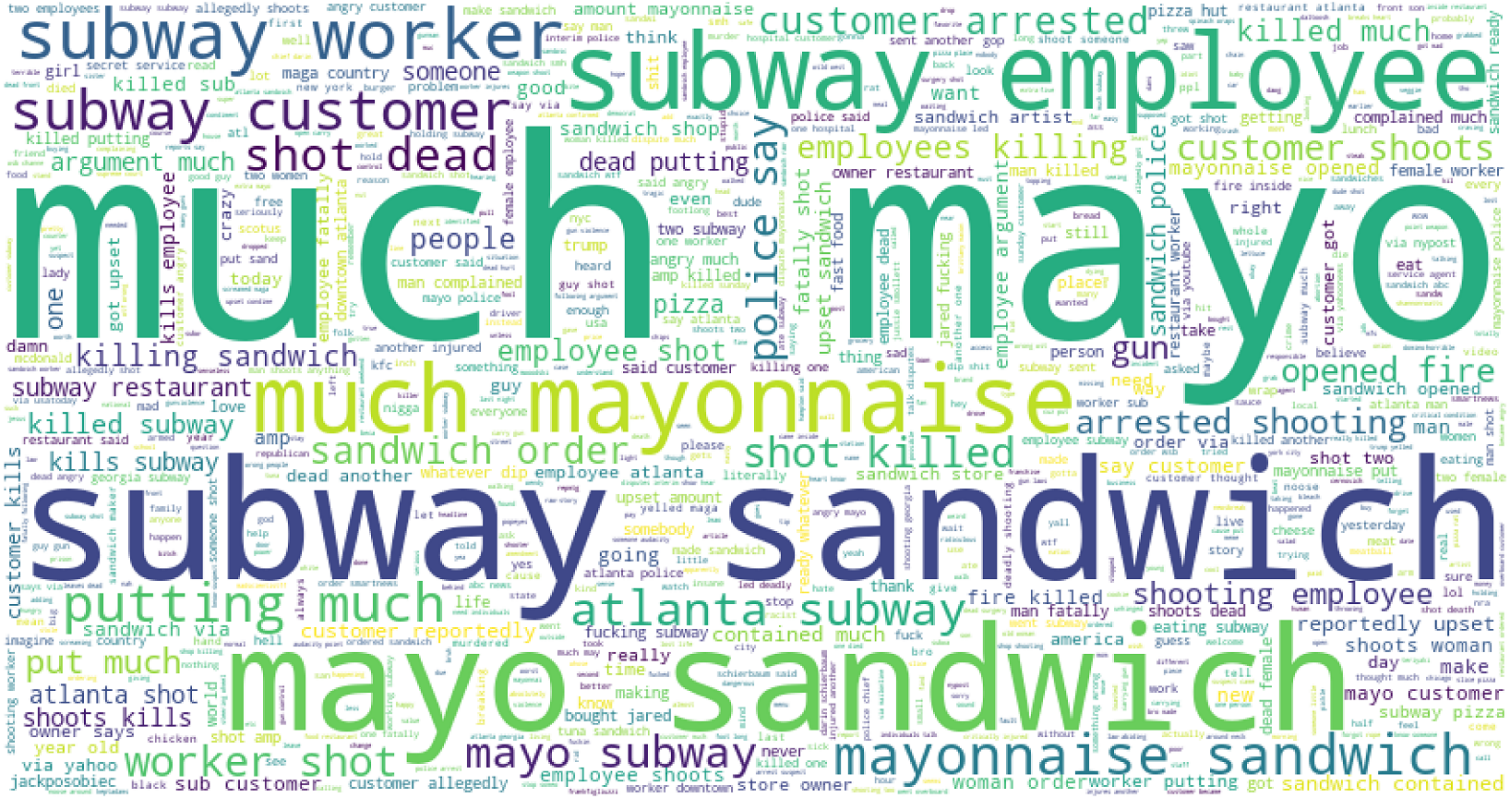
Word cloud for Pizza Hut-related tweets.

Table 15 shows the frequently used negative and positive words in the tweets for Subway food. Topics are extracted using LDA modeling on the preprocessed data. Results show that ‘killed’, ‘complaint’, ‘woman killed’, ‘fucking’, ‘wtf wrong’, ‘shot employee’, and several other highly negative and extremely harsh words are used in negative tweets. The word cloud for Pizza hut is given in Fig. 8.

**Table 15:** Top discussed topics using LDA technique from Subway-related tweets.

**Topic no.**	**Positive keywords**	**Negative keywords**
1	pizza subway make got old good really like love amp	amp subway got argument employee pizza shot atlanta restaurant killed
2	man fatally female shooting got shoots kills restaurant woman subway	sandwich man complained shop mayonnaise police subway opened atlanta say
3	love say subway restaurant pizza sandwich food got man employees	shit subway sandwich like got ready dip bought jared fucking
4	subway pizza good love said allegedly employees fatally woman order	woman killed amp subway police upset shot sandwich know man
5	right like woman subway fatally yahoo dead year mayonnaise love	sandwich subway fucking know like order pizza gun employee wrong
6	food fast subway pizza restaurant sub new sandwich people amp	like sandwich subway wtf wrong mayo upset shot man guy
7	subway sandwich really got like workers pizza make food said	subway pizza sandwich shot killed workers employees jared going complained
8	mayonnaise subway got order right new pizza love make say	killed atlanta subway shot employee customer worker got mayo restaurant
9	good subway amp sandwich sub pizza new say gun putting	subway sandwich employee man worker ready putting shooting gun shoots
10	subway sandwich really got like workers pizza make food said	inside upset ready worker opened mad going killing killed complained

**Figure 9:**
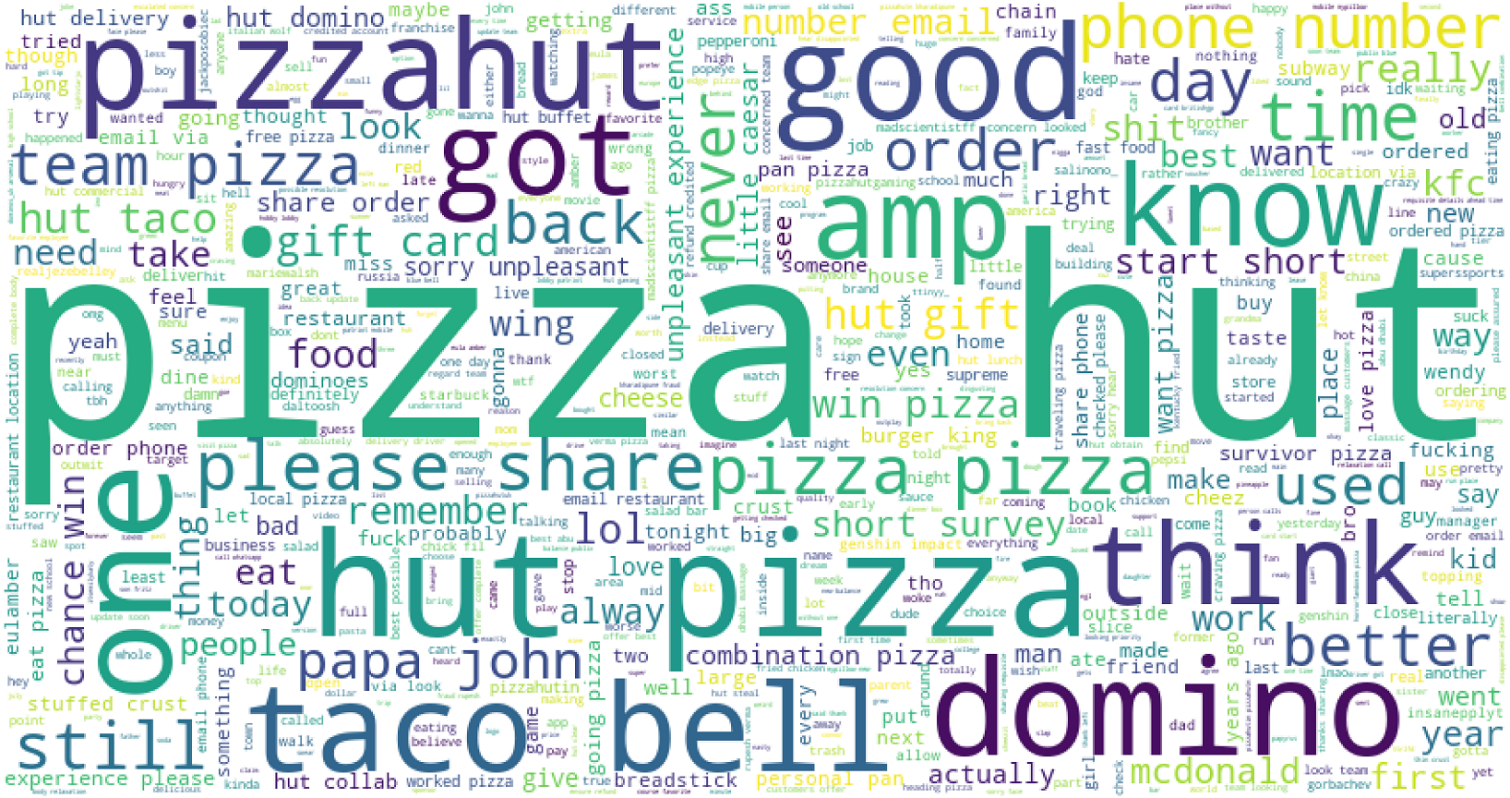
Word cloud for Subway sandwich mayo-related tweets.

Word cloud is used to show how textual information looks, and it is mostly used to analyze data from social platforms (Kabir, Ahmed & Karim, 2020). It is the summary of the whole document, and the word’s size denotes the frequency and prominence of data. It is used to know how much our data is related to research. Figure 9 presents the word cloud for Subway-related tweets.

### Choice of fast food

**Figure 10:**
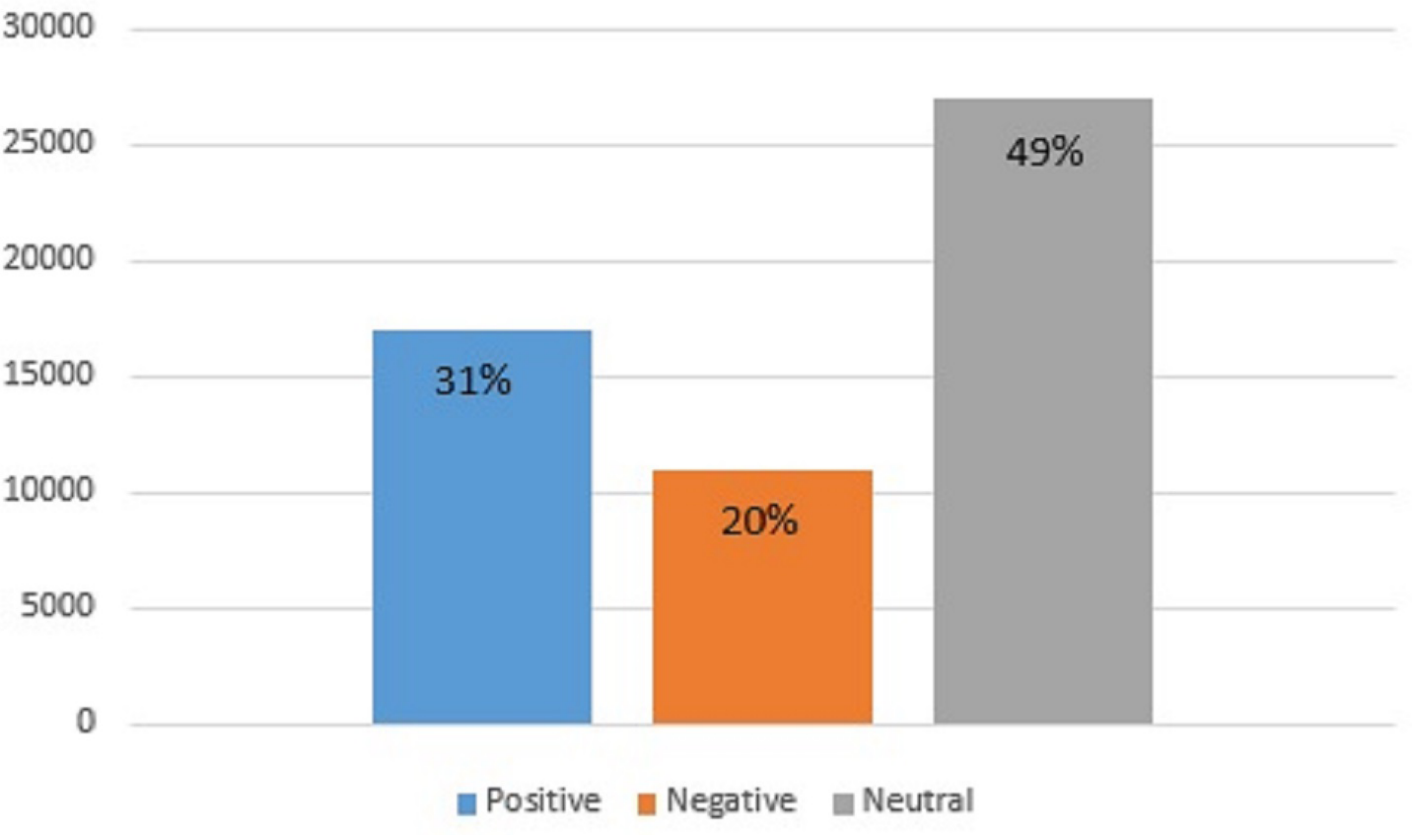
Ratio of positive, negative, and neutral sentiments regarding fast food.

Topic modeling is also carried out to find the sentiments of the people regarding the choice of fast food. For this purpose, the gathered tweets for the considered five companies are taken into account. Results indicate that a higher ratio of people remains neutral regarding selecting fast food with 49% of the total tweets. The people that prefer and like fast food are only 31% which have a positive view of fast food. The remaining 20% do not seem satisfied with fast food and show a negative attitude toward fast food, as shown in Fig. 10.

### Comparison of proposed method with existing studies

We compared the performance of the proposed approach to several state-of-the-art approaches. The findings of the comparison with previously conducted research for sentiment analysis are presented in Table 16. All the results mentioned in Table 16 are taken from only sentiment analysis studies and deep learning models. Srinivas et al. (2021) deployed neural networks and LSTM to analyze the sentiments of tweets. The study achieved 87% accuracy with LSTM. The study employed CNN, NN, and LSTM models. The authors did not use ensemble models to improve accuracy. Similarly, Singh et al. (2022) combined LSTM and RNN models with enhanced attention layers to obtain better performance. The authors used different hyper-parameters and activation layers to increase the model performance and achieved an accuracy of 84.563%. Omara, Mousa & Ismail (2022) conducted a study on sentiment analysis using BiGRU+CNN. In comparison to these studies, the proposed BiLSTM + GRU model performs much better and shows better accuracy.

**Table 16:** Comparison of the proposed method with the state-of-the-art studies.

**Reference**	**Methods**	**Results**	**Published**
Srinivas et al. (2021)	LSTM	87%	2021
Jain, Kumar & Mahanti (2018)	LSTM	43%	2018
Dashtipour et al. (2021)	2D-CNN	89%	2021
Goularas & Kamis (2019)	CNN+LSTM	59%	2019
Abdelgwad (2021)	BERT	89.5%	2021
Singh et al. (2022)	LSTM +RNN	85%	2022
Hassan & Mahmood (2018)	CNN+LSTM	93%	2018
Omara, Mousa & Ismail (2022)	BiGRU+CNN	89%	2022
**Proposed study**	**BiLSTM+GRU**	**95.62%**	**–**

## Conclusion

With the rise of social media platforms and micro-blogging websites, sharing views and comments regarding products and services has become a norm in modern-day society. As a result, people read such views before visiting a mall, restaurant, etc. before ordering something. Such reviews contain important information and automatic analysis of peoples’ sentiments has become important to make better and more informed decisions. This study performs sentiment analysis and topic modeling of the top five fast food companies including KFC, McDonald’s, Burger King, Pizza Hut, and Subway Burger to find the sentiments of the public about these foods. Besides using lexicon-based approaches like TextBlob and VADER, this study employs standalone deep learning models for sentiment classification. Anyhow, the emphasis is placed on using the customized deep ensemble models for the task of sentiment classification into positive, negative, and neutral. Results indicate that deep ensemble models yield better performance than both standalone models and lexicon approaches. The BiLSTM-GRU model performed well and obtained 95.62% accuracy with TextBlob features to classify the tweets. It is also revealed that the highest number of negative sentiments are shown for Subway with high-intensity negative words. Findings also show that 20% of respondents have a negative opinion of fast food, 31% have a positive opinion and 49% remain neutral regarding the food choice, restaurants, service, quality, etc. Sentiment analysis might be of assistance to both the restaurants and their customers in determining the quality of the food, service, and overall experience provided by the restaurants. Nevertheless, this study can further be improved from several aspects. First, the role of data preprocessing approaches can further be investigated. Second new lexicon-based approaches can be utilized for labeling and further experiments can be performed. Lastly, advanced transformers-based approaches can be adopted to analyze their effectiveness for sentiment classification for food reviews.

## Supplemental Information

10.7717/peerj-cs.1193/supp-1 Supplemental Information 1 Code and raw data

## Additional Information and Declarations

### Data Availability

The following information was supplied regarding data availability: The raw data and code are available in the Supplemental FIles.

## References

[ref-1] Abdalla G, Özyurt F (2021). Sentiment analysis of fast food companies with deep learning models. The Computer Journal.

[ref-2] Abdelgwad MM (2021). Arabic aspect based sentiment analysis using BERT.

[ref-3] Ahmed HM, Javed Awan M, Khan NS, Yasin A, Faisal Shehzad HM (2021). Sentiment analysis of online food reviews using big data analytics. Elementary Education Online.

[ref-4] Ali NM, Abd El Hamid MM, Youssif A (2019). Sentiment analysis for movies reviews dataset using deep learning models. International Journal of Data Mining & Knowledge Management Process (IJDKP).

[ref-5] Alshamsi A, Bayari R, Salloum S (2020). Sentiment analysis in English texts. Advances in Science, Technology and Engineering Systems Journal.

[ref-6] Alzahrani ME, Aldhyani TH, Alsubari SN, Althobaiti MM, Fahad A (2022). Developing an intelligent system with deep learning algorithms for sentiment analysis of E-Commerce product reviews. Computational Intelligence and Neuroscience.

[ref-7] Ao S (2018). Sentiment analysis based on financial tweets and market information.

[ref-8] Bindra A (2012). SocialLDA: scalable topic modeling in social networks. Thesis (Master’s).

[ref-9] Bleich SN, Wolfson JA, Jarlenski MP (2017). Calorie changes in large chain restaurants from 2008 to 2015. Preventive Medicine.

[ref-10] Chandrasekaran G, Hemanth J (2022). Deep learning and TextBlob based sentiment analysis for coronavirus (COVID-19) using twitter data. International Journal on Artificial Intelligence Tools.

[ref-11] Chiorrini A, Diamantini C, Mircoli A, Potena D (2021). Emotion and sentiment analysis of tweets using BERT.

[ref-12] Chowdhary K (2020). Natural language processing. Fundamentals of Artificial Intelligence.

[ref-13] Clauson A (2000). Spotlight on national food spending. Food Review/National Food Review.

[ref-14] Dashtipour K, Gogate M, Adeel A, Larijani H, Hussain A (2021). Sentiment analysis of persian movie reviews using deep learning. Entropy.

[ref-15] Devlin J, Chang M-W, Lee K, Toutanova K (2018). Bert: pre-training of deep bidirectional transformers for language understanding.

[ref-16] Dhola K, Saradva M (2021). A comparative evaluation of traditional machine learning and deep learning classification techniques for sentiment analysis.

[ref-17] Elfaik H, Nfaoui EH (2021). Deep attentional bidirectional LSTM for arabic sentiment analysis in twitter.

[ref-18] Endsuy RD (2021). Sentiment analysis between VADER and EDA for the US presidential election 2020 on twitter datasets. Journal of Applied Data Sciences.

[ref-19] Fang X, Zhan J (2015). Sentiment analysis using product review data. Journal of Big Data.

[ref-20] Farkhod A, Abdusalomov A, Makhmudov F, Cho YI (2021). LDA-based topic modeling sentiment analysis using Topic/Document/Sentence (TDS) model. Applied Sciences.

[ref-21] French SA, Harnack L, Jeffery RW (2000). Fast food restaurant use among women in the Pound of Prevention study: dietary, behavioral and demographic correlates. International Journal of Obesity.

[ref-22] Gandhi UD, Malarvizhi Kumar P, Chandra Babu G, Karthick G (2021). Sentiment analysis on twitter data by using convolutional neural network (CNN) and long short term memory (LSTM). Wireless Personal Communications.

[ref-23] García S, Luengo J, Herrera F (2015). Data preprocessing in data mining.

[ref-24] Goularas D, Kamis S (2019). Evaluation of deep learning techniques in sentiment analysis from twitter data.

[ref-25] Hasan MR, Maliha M, Arifuzzaman M (2019). Sentiment analysis with NLP on twitter data.

[ref-26] Hassan A, Mahmood A (2018). Convolutional recurrent deep learning model for sentence classification. Ieee Access.

[ref-27] Hazarika D, Konwar G, Deb S, Bora DJ (2020). Sentiment analysis on twitter by using textblob for natural language processing. International Conference on Research in Management and Technovation.

[ref-28] Hossin M, Sulaiman MN (2015). A review on evaluation metrics for data classification evaluations. International Journal of Data Mining & Knowledge Management Process.

[ref-29] Jain VK, Kumar S, Mahanti P (2018). Sentiment recognition in customer reviews using deep learning. International Journal of Enterprise Information Systems (IJEIS).

[ref-30] Jelodar H, Wang Y, Yuan C, Feng X, Jiang X, Li Y, Zhao L (2019). Latent Dirichlet allocation (LDA) and topic modeling: models, applications, a survey. Multimedia Tools and Applications.

[ref-31] Kabir AI, Ahmed K, Karim R (2020). Word cloud and sentiment analysis of amazon earphones reviews with R programming language. Informatica Economica.

[ref-32] Karimi A, Rossi L, Prati A (2021). Adversarial training for aspect-based sentiment analysis with bert.

[ref-33] Kuang X, Chae H, Hughes B, Natriello G (2021). An LDA topic model and social network analysis of a school blogging platform.

[ref-34] Kydros D, Argyropoulou M, Vrana V (2021). A content and sentiment analysis of Greek tweets during the pandemic. Sustainability.

[ref-35] Maier D, Waldherr A, Miltner P, Wiedemann G, Niekler A, Keinert A, Pfetsch B, Heyer G, Reber U, Häussler T (2018). Applying LDA topic modeling in communication research: Toward a valid and reliable methodology. Communication Methods and Measures.

[ref-36] Meera S, Geerthik S (2022). Natural language processing. Artificial Intelligent Techniques for Wireless Communication and Networking.

[ref-37] Mujahid M, Lee E, Rustam F, Washington PB, Ullah S, Reshi AA, Ashraf I (2021). Sentiment analysis and topic modeling on tweets about online education during COVID-19. Applied Sciences.

[ref-38] Naskar D, Mokaddem S, Rebollo M, Onaindia E (2016). Sentiment analysis in social networks through topic modeling.

[ref-39] Omara E, Mousa M, Ismail N (2022). Character gated recurrent neural networks for Arabic sentiment analysis. Scientific Reports.

[ref-40] Onan A (2021). Sentiment analysis on massive open online course evaluations: a text mining and deep learning approach. Computer Applications in Engineering Education.

[ref-41] Pang B, Lee L (2008). Opinion mining and sentiment analysis. Foundations and Trends in Information Retrieval.

[ref-42] Pokharel BP (2020). Twitter sentiment analysis during covid-19 outbreak in Nepal. https://ssrn.com/abstract=3624719.

[ref-43] Pota M, Ventura M, Fujita H, Esposito M (2021). Multilingual evaluation of pre-processing for BERT-based sentiment analysis of tweets. Expert Systems with Applications.

[ref-44] Prastyo PH, Sumi AS, Dian AW, Permanasari AE (2020). Tweets responding to the Indonesian Governments handling of COVID-19: sentiment analysis using SVM with normalized poly kernel. Journal of Information Systems Engineering and Business Intelligence.

[ref-45] Prottasha NJ, Sami AA, Kowsher M, Murad SA, Bairagi AK, Masud M, Baz M (2022). Transfer learning for sentiment analysis using BERT based supervised fine-tuning. Sensors.

[ref-46] Ramadhani AM, Goo HS (2017). Twitter sentiment analysis using deep learning methods.

[ref-47] Rani S, Kumar P (2019). Deep learning based sentiment analysis using convolution neural network. Arabian Journal for Science and Engineering.

[ref-48] Raza MR, Hussain W, Merigó JM (2021). Cloud sentiment accuracy comparison using RNN, LSTM and GRU.

[ref-49] Rehman AU, Malik AK, Raza B, Ali W (2019). A hybrid CNN-LSTM model for improving accuracy of movie reviews sentiment analysis. Multimedia Tools and Applications.

[ref-50] Rokach L (2009). Pattern classification using ensemble methods.

[ref-51] Rupapara V, Rustam F, Amaar A, Washington PB, Lee E, Ashraf I (2021). Deepfake tweets classification using stacked Bi-LSTM and words embedding. PeerJ Computer Science.

[ref-52] Rydell SA, Harnack LJ, Oakes JM, Story M, Jeffery RW, French SA (2008). Why eat at fast-food restaurants: reported reasons among frequent consumers. Journal of the American Dietetic Association.

[ref-53] Sabba S, Chekired N, Katab H, Chekkai N, Chalbi M (2022). Sentiment Analysis for IMDb Reviews Using Deep Learning Classifier.

[ref-54] Saif H, He Y, Alani H (2012). Semantic sentiment analysis of twitter.

[ref-55] Singh C, Imam T, Wibowo S, Grandhi S (2022). A deep learning approach for sentiment analysis of COVID-19 reviews. Applied Sciences.

[ref-56] Singh M, Jakhar AK, Pandey S (2021). Sentiment analysis on the impact of coronavirus in social life using the BERT model. Social Network Analysis and Mining.

[ref-57] Srinivas ACMV, Satyanarayana C, Divakar C, Sirisha KP (2021). Sentiment analysis using neural network and LSTM.

[ref-58] Trivedi SK, Singh A (2021). Twitter sentiment analysis of app based online food delivery companies. Global Knowledge, Memory and Communication.

[ref-59] Xu G, Meng Y, Qiu X, Yu Z, Wu X (2019). Sentiment analysis of comment texts based on BiLSTM. IEEE Access.

[ref-60] Yang S, Zhang H (2018). Text mining of Twitter data using a latent Dirichlet allocation topic model and sentiment analysis. International Journal of Computer and Information Engineering.

[ref-61] Yenter A, Verma A (2017). Deep CNN-LSTM with combined kernels from multiple branches for IMDb review sentiment analysis.

